# B5GEMINI: AI-Driven Network Digital Twin

**DOI:** 10.3390/s22114106

**Published:** 2022-05-28

**Authors:** Alberto Mozo, Amit Karamchandani, Sandra Gómez-Canaval, Mario Sanz, Jose Ignacio Moreno, Antonio Pastor

**Affiliations:** 1 ETSI Sistemas Informáticos, Dpto. Sistemas Informáticos, Universidad Politécnica de Madrid, 28031 Madrid, Spain; amit.kbatra@alumnos.upm.es (A.K.); sm.gomez@upm.es (S.G.-C.); antonio.pastorperales@telefonica.com (A.P.); 2 ETSI Telecomunicación, Dpto. Ingeniería de Sistemas Telemáticos, Universidad Politécnica de Madrid, 28040 Madrid, Spain; mario.sanz@upm.es (M.S.); joseignacio.moreno@upm.es (J.I.M.); 3 Telefónica I+D., 28050 Madrid, Spain

**Keywords:** digital twin, network digital twin, artificial intelligence, machine learning, telecommunications

## Abstract

Network Digital Twin (NDT) is a new technology that builds on the concept of Digital Twins (DT) to create a virtual representation of the physical objects of a telecommunications network. NDT bridges physical and virtual spaces to enable coordination and synchronization of physical parts while eliminating the need to directly interact with them. There is broad consensus that Artificial Intelligence (AI) and Machine Learning (ML) are among the key enablers to this technology. In this work, we present B5GEMINI, which is an NDT for 5G and beyond networks that makes an extensive use of AI and ML. First, we present the infrastructural and architectural components that support B5GEMINI. Next, we explore four paradigmatic applications where AI/ML can leverage B5GEMINI for building new AI-powered applications. In addition, we identify the main components of the AI ecosystem of B5GEMINI, outlining emerging research trends and identifying the open challenges that must be solved along the way. Finally, we present two relevant use cases in the application of NDTs with an extensive use of ML. The first use case lays in the cybersecurity domain and proposes the use of B5GEMINI to facilitate the design of ML-based attack detectors and the second addresses the design of energy efficient ML components and introduces the modular development of NDTs adopting the Digital Map concept as a novelty.

## 1. Introduction

The concept and development of the Digital Twin (DT) was first formulated by Grieves and Vickers [1]. According to that original definition, a DT is a virtual model that resembles the characteristics and behavior of a physical asset or system, modeling its components and properties, as well as the interactions of the entity with the environment. A DT can be used to monitor and manage a physical asset or system in real-time or use it to recreate its behavior in predefined virtual scenarios in order to define a better industrial process or detect problems before the real implementation.

The application of the DT concept in the field of communication networks has also recently gained attention in both the research community and the industrial sector, leading to the emergence of the Network Digital Twin (NDT) concept. An NDT is a virtual representation of a telecommunications network that accurately models the devices, communication links, operating environment, and applications running on the network. NDTs are a new way of designing and managing networks, in which multiple physical assets and their corresponding virtual twins are connected together to share information and collaborate to complete a set of tasks [2]. In NDTs, network operators in coordination with service providers and telecommunication equipment suppliers can validate new functionalities prior to their incorporation into the network or emulate existing situations in the network and analyze the effect of applying different policies in the network.

By leveraging network data, NDTs can be used to build a virtual representation of a network. The data should replicate the expected behavior of the network; for this reason, the most common source are live feeds from the real network, although data collected from testbeds or simulations that reflect the intended behavior can also be used.

By replicating different environments in a lab and running multiple scenarios, NDTs offer a cost-effective way to assess performance, predict the impact of environmental changes (such as cyber threats), and optimize network processes and decision making accordingly.

Although NDTs are still in their nascent stage, it is envisioned that they will have a pervasive impact on the telecommunication industry, and they are pictured to be a keystone technology for the fourth industrial revolution. The pursue of this interest has led to an accelerated research effort in the last years, mainly because they can be used to thoroughly dissect the response of a network under different conditions and test new networking designs without compromising the safety of the physical network. Additionally, by creating an NDT, the real time optimization of its corresponding physical equivalent is also possible. Furthermore, NDTs can help secure traditional networks by enabling fast identification and isolation of network failures to quickly respond to security threats.

An NDT can be interpreted as a black-box model of a network that can be parameterized in multiple ways to recreate different network scenarios (e.g., different topologies, traffic volume, dynamic placement policies); by adjusting the parameter values, it is possible to observe the resulting changes in the NDT in real time and evaluate the situation using different metrics defined according to the operator’s needs. More interestingly, the above is possible without disrupting the real network or incurring the construction of costly test environments.

In search of mechanisms to improve network intelligence, the use of artificial intelligence (AI) for the creation and operation of NDTs has begun to be explored in recent years. In fact, there is broad consensus that AI and ML are among the key enablers of the NDT concept. AI methods can be used to improve the accuracy of NDT simulations and to enable new services that ultimately improve user experience, such as self-healing networks, autonomous network management and security monitoring. In addition, AI methods can also be used to automatically learn and adapt to changes in real assets. This can help ensure that nondestructive testing remains accurate over time.

Communication networks often work with large amounts of heterogeneous data that differ greatly in format and content. For this reason, creating a model of the network that accurately represents its behavior is a highly challenging task. Fortunately, Machine Learning (ML) techniques can help to extract insight from the real network data and construct the corresponding NDT of the network automatically [3]. Furthermore, the increasing excitement generated by the NDT concept in the last few years has raised interest in the NMRG IETF standardization group. The NMRG group has recently published a draft that proposes a reference framework for NDTs and their possible applications [4]. Among these applications, ML model training and validation is explicitly mentioned. It should also be emphasized that one of the main concepts in this draft is the recommendation to have an exchange of information between the real physical network and the DT to obtain maximum benefit.

In order to achieve the goals proposed by the IETF draft, in this work, we make the case of ML methods as a key enabler for the construction of an NDT that models complex 5G and beyond networks. To that end, we present in this work B5GEMINI, a proposal for an AI-based NDT architecture, whose main objective is to deploy a 5G/6G NDT support platform capable of building a specific network configuration and generating the necessary traffic to be subsequently used for different analysis activities focused on advanced scenarios, such as cybersecurity and network management, which range from resource optimization to security enforcement. It should be noted that B5GEMINI is an acronym for “Beyond 5G GEMINI”, where the term “gemini” corresponds to the Latin word for “twins”. Due to the inherent complexity of 5G/6G models, B5GEMINI aims to make extensive use of AI to provide them with anticipatory and self-learning capabilities. 

B5GEMINI addresses several complementary objectives: the development of a 5G/6G NDT infrastructure and the design and integration of advanced AI components and use cases in the NDT for realistic experimentation. It is worth noting that current NDT proposals tend to apply AI to add intelligence in different ways to network orchestration processes. B5GEMINI goes one step beyond and also focuses on the use of NDT for training and validating AI/ML components in a controlled scenario when potentially harmful situations could occur if trained or tested in a real network. B5GEMINI will provide a virtualized representation of the 5G/6G network meant to analyze, diagnose, emulate, and control the physical network. The virtual system will start with a simple form of the real system and, using AI self-learning capabilities and data updating, evolve gradually to a more realistic image. With the support of AI, complex data sources and telemetry (e.g., IoT sensors, network data) will be fed into sophisticated data interpretation processes to facilitate the replica of 5G/6G network components. In addition, B5GEMINI will allow for the deploying of complex network scenarios in a controlled way, launching clients and servers and collecting the traffic generated by them even if they interact with clients and servers outside the NDTs, typically on the wider Internet.

B5GEMINI will enable an extensive use of advanced AI mechanisms to realize several valuable AI applications, such as (i) the training and testing of ML components to deploy smart applications such as cybersecurity or network management in real-time environments; (ii) the development of intelligent support to management, orchestration, and dynamic control of the network components (e.g., Virtual Network Functions—VNFs, Network Services—NS and Slices); (iii) the use of the NDT as a platform to perform distributed training and inference processes for ML and DL models using on-demand GPU virtualization; (iv) ML as a service to deploy off-the-shelf pre-trained ML models in the NDT allocated resources to perform ML tasks with a high degree of efficiency, avoiding costly training and testing tasks; (v) the use of the NDT for network deployment planning. In addition, B5GEMINI will allow one to realize several interesting use cases, such as (i) the design of ML components for network cybersecurity capabilities and (ii) the leverage NDT as a validation environment for energy consumption optimization of the ML/DL components deployed in the real network. It is worth noting that other types of applications, such as cyber-ranges or educational applications in general, could greatly benefit from the use of a realistic and controlled 5G/6G network environment such as B5GEMINI, even if only the virtual twin is used.

Finally, and regarding that the time and cost of planning, designing, and implementing a complete NDT system from scratch can be unaffordable, B5GEMINI includes in its design a modular building approach based on the Digital Map concept, which is a novel concept that has appeared in recent IETF discussions as a possible way to specifically solve this problem.

The remainder of this work is organized as follows: Section 2 reviews the literature on the application of AI methods in NDTs and presents the state-of-the-art in the field. Section 3 describes the B5GEMINI architecture, providing details of the underlaying infrastructure and the integration of the 5G core capabilities in it. Section 4 details several interesting applications that B5GEMINI can address, and Section 5 presents the main B5GEMINI AI components jointly with the open challenges associated with their deployment. Section 6 presents two notable use cases related to cyber-security and energy efficiency. In addition, the second use case depicts the application of the novel concept of Digital Maps for a modular NDT construction. Finally, Section 7 summarizes the main findings of this work.

## 2. Related Work

The concept of DT as a virtual copy associated with a physical entity was introduced in 2003 by Michael Grieves at a Product Lifecycle Management (PLM) conference [5]. NASA was an early adopter of the concept, starting to use DTs to monitor spacecraft health since 2010. However, the term “digital twin” was first coined in 2011 by John Vickers. In practical terms, the first DT was developed by Tuegel et al. [6] to predict the structural integrity of next-generation fighter aircrafts over their lifetime. The concept began gaining traction in 2015 due to the falling costs of sensors, processors and data storage, and the increasing availability of broadband wireless connectivity, leading to the so-called “IoT revolution” [7].

The exchange of information between a physical object and its DT is a crucial element for the creation of a successful DT: the virtual twin must quickly adapt to changes in its physical counterpart, just as the physical object must promptly respond to interventions taking place in the virtual twin. This transfer of information from the physical environment to its virtual twin is made possible using communication networks that connect the two parties. NDT result from the virtualization of the communications infrastructure of a real network, allowing the interconnection of DTs that are part of a complete system as well as supporting the bidirectional data flow with the real network. Thanks to this two-way communication, NDT is expected to enable a new wave of AI-powered applications that can more accurately predict outcomes and make better decisions to support network operators in the deployment and operation procedures involved in network management. In this section, we review the most interesting applications that we discovered in our research. 

At first, we observed considerable research effort leveraging this technology towards the fulfillment of the expectations placed on 5G and beyond communications. The potential of edge and cloud computing platforms to manage DT-related data should be considered. In particular, edge computing allows for the leveraging of computing capabilities of the distributed nodes at the edge of the network, allowing one to reduce the bandwidth and dependency with the cloud gateway [8]. There is a large body of work focused on providing solutions to further minimize network and data processing latency between end users and edge servers to facilitate the adoption of intelligence-driven applications. In our research, we have observed a particular interest in looking for the most optimal ways to place and migrate DT in a network that combines these two paradigms [9], i.e., an NDT optimization task in which ML and DL techniques emerge as the most attractive solution. They propose a new approach based on DRL to find the optimal placement for DTs in order to reduce system latency. This algorithm takes into account both the placement strategy and the mobility of users. In addition, they suggest using transfer learning to migrate DTs to new users.

On the same line, Lu et al. proposed to combine DT with edge computing to efficiently connect IoT devices with CPS in an Industrial IoT scenario (IIoT) [9,10,11]. More specifically, they propose to model DTs of the physical devices present in the user layer and place them in the edge to allow their operation from the CPS to achieve a better optimization of the manufacturing process. For this purpose, they propose an asynchronous federated learning scheme to build the DT of those devices using ML techniques. In federated learning, different devices in a network collaborate to train a shared model. This can be conducted by dividing the dataset among the devices, and each device trains its own model. These individual models can then be merged to create a single, more powerful model. Federated learning allows data collected by different entities to be exploited, eliminating the need for a large, centralized ML infrastructure. This can be important for organizations that need to maintain the confidentiality of their data or have limited resources. Leveraging this concept, Lu et al. build their architecture based on this scheme to alleviate privacy concerns in NDT, while reducing data transfer overhead.

To minimize energy consumption and reduce data transfers in a Mobile Edge Computing (MEC) scenario, Dong et al. propose an optimization of resource allocation and data offloading probabilities to the cloud, as well as an efficient mapping of the association between users and edge devices, by approximating these strategies using DL models in a supervised manner [12]. For this purpose, an NDT of the communication network is constructed and deployed on a central server, different configurations are evaluated in the NDT, and the best ones are used to train a DNN model in an off-line fashion.

Groshev et al. describes the key technologies that will support the deployment and operation of an NDT in an industrial setting, including edge and fog computing, and a 5G network [13]. Additionally, they determine the role of AI methods to address some of the challenges posed in this scenario. More interestingly, they identify AI as a tool to optimally allocate and automatically scale computing resources for an NDT to satisfy key performance indicators (KPIs) such as latency and security requirements. In addition, they conduct an experimental evaluation as a proof-of-concept to predict the movement of a robotic arm using data collected from a DT to train a variety of ML models that are subsequently compared.

A vehicular network (VN) is a collection of nodes that are connected by links. Nodes can be cars, trucks, buses, or other vehicles. The links can be streets, highways, or other transportation infrastructure. A VN can be used to model the movement of vehicles in a city. A software-defined vehicular network (SDVN) is a VN that uses the concept of software-defined networking (SDN) to allow network administrators to manage network traffic through software defined rules instead of hardware configurations [14]. This enables large networks to be managed more efficiently and to respond quickly to changing network conditions. Based on this concept, Zhao et al. propose to create an NDT of a SDVN as a simulation environment to evaluate the quality and reliability of ML and DL models before applying them in the physical network [15]. This simulations environment, which they call the “Intelligent Digital Twin” (IDT), allows one to assess whether the models remain robust to the intrinsic dynamic changes of a VN and retrains the models if needed.

A similar use case is presented in [3]. In this paper, the authors identify 5G as a key technology to enable efficient inter-vehicle communication to satisfy the necessary low latency and high throughput requirements that are critical requirements to achieve autonomous driving. In this particular context, with the objective of improving road safety and traffic management, the Spirent team proposes to achieve this goal by creating a Digital Twin of a 5G SDVN to emulate all aspects of a 5G network and its interactions with vehicles under different realistic scenarios (e.g., congestion and vehicle density) in a controlled manner [16]. In this approach, AI methods can be introduced to understand and evaluate vehicle behavior, optimize vehicle-to-vehicle communication, and enable end-to-end validation of the entire 5G SDVN. In this way, the built NDT serves as a testbed to verify vehicle performance prior to deployment in a real-world environment.

Similar to the above presented case, in 2016, General Electric filed a US patent for a wind farm management solution that leverages the concept of NDT to facilitate the operations and test diverse strategies in a simulation environment powered by AI [17]. The proposal is composed of two communication networks, a local network that connects individual wind turbines with the control system, and a cloud-based communication network that allows operators to configure and operate the wind farm remotely via the Internet. The proposed solution includes ML models to analyze the data generated by the DT to recommend actions and simulate them on the provided graphical user interface (GUI) for continuous feedback. ML techniques are trained on real data and integrated in the NDT. The developed models are then used to assist operators in the management of the wind turbines through the GUI, offering automated suggestions and allowing one to simulate the selected strategies in the NDT to quickly obtain feedback.

From a more creative point of view, there have been several interesting proposals that attempt to determine other roles that ML can play within NDT. One of those interesting use cases of ML in the operation of NDT is presented in [2]. As noted by the authors, the behavior of the virtual twin is expected not to disturb the physical space. To that end, ML techniques could be used to validate the next actions of the real system using data generated from the NDT model to prevent unwanted actions in the physical environment. In this scenario, which is also envisaged in [3,18], ML would serve as an anomaly detection tool for network operators, enabling them to quickly anticipate abnormal situations and mitigate further damage.

Nguyen et al. anticipated 5G-based NDT technology as a city management service that can provide situational awareness and decision-making for city managers and first responders [3]. In particular, they present the case for NDT as a ML-powered tool for the prediction of infectious disease outbreaks. More specifically, they propose to create an NDT that represents the city infrastructure and the overlaying 5G network. With the increased bandwidth and low latency that 5G networks provide, it will be possible to collect and analyze data from a large number of sensors in real time to track people movement, activities and interactions. Combining this information with past and current epidemiological data and using a combination of ML and conventional data analytic processes, it is possible to predict the most likely outbreak scenarios to occur. In this way, public health officials and other related professionals can be better prepared to develop strategies to respond to these situations more effectively. Extending this idea, the combination of 5G-based DT and ML can be applied to the development of more efficient and ambitious smart city applications [19], such as intelligent transportation, smart energy, and intelligent security.

We have also identified some works where DT-based systems are developed with the help of ML techniques [20,21]. ML and DL methods are also expected to play an important role in NDT model building, as also anticipated in [18], but, to this day, there is a need for further systematic research in this area to find the most suitable methods and models for different types of data and to determine how these models will interoperate with the rest of the elements and contribute to the NDT.

In summary, there is a lack of studies on the design and development of NDT and, in particular, on the integration of ML and DL methods in such systems. The research proposed in this article aims to fill this gap and contribute to the advancement of this area by providing B5GEMINI, an architecture for NDTs that considers AI components by design. Furthermore, all the articles we have collected for this research approach the design and construction of NDTs from a holistic perspective, aiming to implement all their components and interconnections in a complete development cycle to achieve the desired functionality, which requires extensive and careful planning and implies high risk in the development process to achieve a successful and sustainable outcome. As a novelty, we introduce the Digital Map (DMap) concept in B5GEMINI, justified on the need to build a complete NDT in a modular way and thus avoid the cost of planning, designing, and implementing a complete NDT system from scratch. Furthermore, B5GEMINI proposes as novelty the NDT-ready label to identify physical networks and NDTs that are fully interoperable by means of a standardized communication channel. In addition, we also provide fundamental AI solutions that can be integrated into an NDT and identify key AI components that can be included in B5GEMINI architecture to provide an NDT with intelligent capabilities that facilitate network operations or enable new use cases. Finally, two interesting use cases are proposed to show the adequacy of the B5GEMINI proposal: The design of a ML-based cryptomining detector for cyber-security solutions and the optimization of AI/ML components reduces its energy consumption both in training and inference phases.

## 3. B5GEMINI Network Digital Twin

In this section, we first describe Mouseworld Lab, the origin of B5GEMINI, as it contains some of the core components of the DTN architecture that form the foundation of B5GEMINI. Then, we explain the infrastructure of B5GEMINI and all its related components, with special emphasis on the integration of the 5G Core in B5GEMINI. Last, we introduce a novel approach for building modular NDT based on the Digital Map (DMap) concept.

### 3.1. Mouseworld

**Figure 1:**
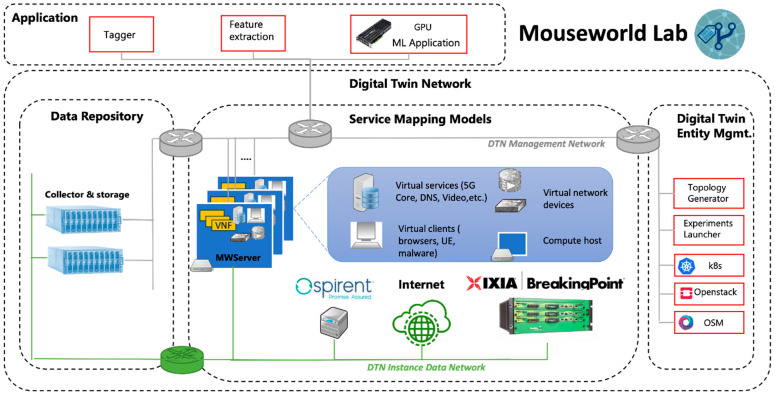
Mouseworld Lab.

One of the corner stones of B5GEMINI is the Mouseworld Lab [22], a controlled environment set up in the Telefónica I+D premises for running experiments that allow deploying complex network scenarios in a controlled way and generate realistic labeled data sets for training supervised ML components and validate supervised and unsupervised solutions. The Mouseworld Lab provides a way to launch clients and servers, and collects the traffic generated by them even if they interact with clients and servers outside the Mouseworld Lab on the Internet; finally, it adds labels to this traffic without operator intervention. This environment is deployed on an NFV-enabled architecture, under the management of an orchestrator (NFVO), extending an ETSI NFV MANO stack as necessary (Figure 1).

AI and ML were included by design in the Mouseworld Lab to allow the deployment of realistic network management and cybersecurity use cases where ML could be seamlessly trained, tested, and deployed. Therefore, the Machine Learning Orchestration is part of the Digital Twin Entity Management and is composed of a Topology Generator and an Experiment Launcher. The first uses predefined templates, interacts with OSM (ETSI NFV SOL-005 interface) and OpenStack (Glance Image service API) provisioning NSD (Network Service Descriptors) and VNFD (VNF descriptors) compatible with OSM, detailing the network functions, the links, and their day-1 configurations. The Experiment Launcher makes day-2 configurations and triggers the emulations functions for dataset generation, and it is described with OSM’s ProxyCharms or with dedicated scripts. The current version uses a configuration file to define the statistical distribution of the traffic, the number of intervals in which the experiment is divided and the duration, and the type of service that it is emulating. Currently, there are defined three scenarios: traffic classification, crypto mining malware detection, and DoH attacks. 

### 3.2. Introduction to Network Digital Twinning

In the digital twinning context, two concepts with differentiated characteristics are established [2]:

1. Digital Twin (DT): Treated as an intelligent system in charge of modeling all the characteristics of a specific physical component. In the case of the DT, the communication performs a one-to-one mapping between the physical twin and its DT, allowing bidirectional feedback between both. From this point of view, using a bidirectional data flow between both worlds, the DT is able to continuously adapt to operational changes based on real-time data and information coming from the physical twin, being able, among other things, to monitor and even predict the future state of the physical twin. In addition, the DT can also be manipulated, and changes made to it can be automatically transferred to the physical twin.
2. NDT (Network Digital Twin): Treated as an intelligent system composed of multiple individual DT systems that are able to model complex interactions. The DTs are interconnected through an abstraction layer, which is in charge of the data flow between the different DTs. The abstraction layer built on top of the actual network functions as an interface capable of extracting and translating data from one DT to another, creating a communication channel between them, and enabling the integration and interoperability of each DT system. As such, the NDT also comprises the infrastructure of the network and the data flow between the different DT systems. In this case, the data flow follows a many-to-many method, interconnecting all DTs with each other within the NDT. This facilitates the exchange of information between all the DTs included in the NDT, achieving more accurate detections of the network state, enriching the real-time analysis and improving decision making about the physical twins.

### 3.3. B5GEMINI Infrastructure

**Figure 2:**
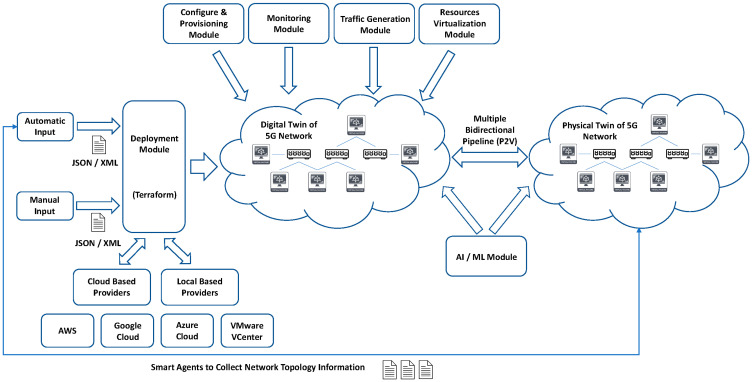
B5GEMINI architecture diagram.

With the aim to design a modular and scalable system for the creation of NDT applied to 5G networks that attempts to model each of the elements of the 5G networks in the form of a DT, we propose B5GEMINI as an evolution of Mouseworld, establishing a complete NDT model capable of accurately emulating the behavior of a 5G network in certain use cases, which will make use of the NDT to be able to apply the task-based on AI. One of the crucial elements that differentiates B5GEMINI from Mouseworld is the two-way communication capability with the real network, which allows real-time synchronization between the real and virtual networks. That is, the configuration of the real network can be replicated in the virtual twin in real-time and the optimizations applied to the virtual twin can be seamlessly deployed in the real network. Figure 2 shows the conceptual design of this complete system. Next, each of the modules that make up the entire system will be reviewed.

#### 3.3.1. System Input for DT Generation

In the phase of creation of the different DTs that will make up the NDT, two general system inputs are stablished: automatic entry and manual entry.

1. Automatic Entry: This is defined with the aim of obtaining an exact replica of the physical object to be modeled, the development of smart agents that can be deployed in the target network, which are in charge of collecting all the information necessary for the DT generation (topological information, hardware and software information, states, etc.). This information is introduced to the deployment module in the JSON or XML format.
2. Manual Input: In the case of not having sufficient permissions to be able to deploy the intelligent agents described in the previous point, an option for loading these files manually by the operators is enabled, avoiding limiting the emulation capacity of the system.

#### 3.3.2. Deployment Module

**Figure 3:**
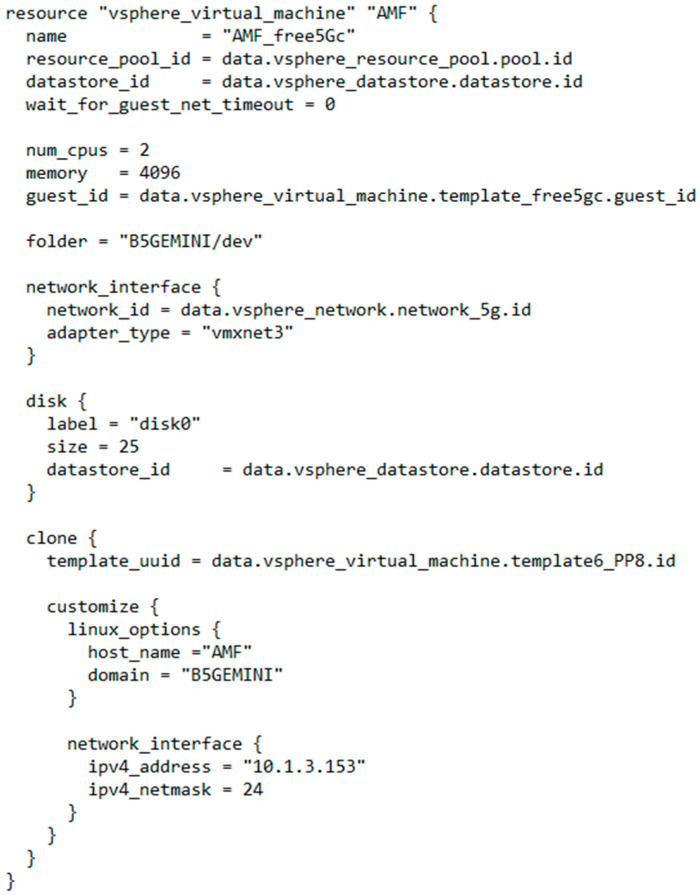
Example of deployment module for a specific resource via Terraform.

Once the initial phase of collecting information for the generation of the DTs is finished, the implementation module is used, which, through the Terraform IaaS (Infrastructure as a Service) [23] tool, will oversee the implementation of the virtual infrastructure that will host the DTs. This module allows, through different providers, one to deploy the infrastructure in any available cloud (AWS, Google Cloud, Azure, IBM, etc.) or in its own virtualization infrastructures, using managers such as vSphere for VCenter (VMWare). Figure 3 shows an example of virtual machine provisioning associated with the 5G AMF element through the Terraform software using the vSphere provider. The main advantage of this technology is the abstraction and non-dependence on specific API for each cloud, since once the Terraform deployment file is generated, it is easily portable to any other cloud that offers a provider compatible with it. Added to the advantage of deployment over any type of infrastructure, is the resource management offered by Terraform, allowing the creation of multiple NDT deployments, isolated from each other, as well as their subsequent elimination, both at the full deployment level and for specific resources within a deployment. Finally, the deployment module makes use of a wide library of virtual machines and Docker [24] containers, which will serve as the basis for the provisioning and configuration of the different DTs to be implemented using technologies such as Kubernetes [25], Docker Swarm [26] or Docker Compose [27]. 

The NFs of the 5G core network are implemented using Docker containers, being able to deploy all the NFs in the same instance or deploy the NFs in a distributed manner, using the Docker bridge network for internal communications between each module or defining the Docker networks’ overlay, respectively. Additionally, in the deployment phase, the docker interfaces are duplicated, allowing the capture of all traffic (signaling and user traffic) that is shared outside and inside the core network.

**Figure 4:**
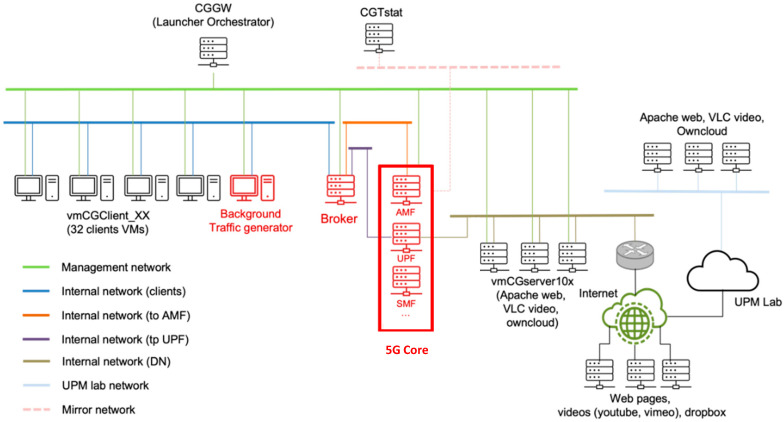
5G core infrastructure and traffic injection applied to B5GEMINI.

Figure 4 shows the integration of the NDT 5G Core at Mouseworld Lab. In this case, Mouseworld Lab is used to apply real context to the DT 5G Core, since it is made up of several virtual devices that act as clients or servers for different types of traffic (streaming video, web pages, etc.). It also has an orchestrator that communicates with all DT devices within the NDT through a management network and the ability to monitor and capture traffic for analysis, statistics, and data set generation. It additionally allows the connection of the NDT to remote services through the Internet.

B5GEMINI devices added to existing infrastructure are marked in red in Figure 4. As can be observed in the figure, the 5G core infrastructure has been integrated into a single instance, using Docker containers, as explained above. However, B5GEMINI allows one to establish an NDT with the different distributed 5G Core NFVs. To allow communication between the different DTs, virtual links are established to communicate the required containers (specifically the AMF and UPF) with other devices in the environment. UPF is connected to an internal network that allows user traffic to reach local or remote servers via the Internet.

On the other hand, the traffic injection module is implemented as a virtual element within the NDT, which can communicate with the AMF through signaling traffic and can receive traffic from client users in the Mouseworld Lab environment. The traffic is piped and sent over the link connected to the UPF container in the dockerized 5G core.

#### 3.3.3. Digital Twin Configuration and Provisioning Module

After completing the infrastructure deployment phase, the configuration and provisioning module is responsible, based on the information collected by the smart agents, for modeling each of the DT and configuring the necessary interconnections to fully simulate the target 5G network. The 5G infrastructure used in the Machine Learning Lab is based on the virtualization of the 5G core network. The network functions (NF) that make up the core interact in a virtual environment within the NDT B5GEMINI infrastructure. The implementation of NFs, used in the NDT B5GEMINI, are based on the free5GC project [28]. This project offers an open implementation of the main NFs needed for core operation. The implemented NFs are:

- Access and Mobility Function (AMF): Implements the control plane function in the core network, including registration, reachability, connection, and mobility management. It uses the NGAP protocol for signaling communication with the RAN nodes.
- Authentication Server Function (AUSF): Provides authentication support for 5G services, handling identifiers, managing authentication, and maintaining session states.
- Non-3GPP Interworking Function (N3IWF): Adds the functionality required for interworking between untrusted non-3GPP networks and the core.
- Network Repository Function (NRF): Is a centralized repository for the service discovery broker for NFs.
- Network Slice Selection Function (NSSF): Used by AMF to select network slice instances for particular services.
- Policy Control Function (PCF): In charge of controlling QoS and control plane policy rules (slicing, roaming, mobility). It also provides the management of subscribers, applications, and network resources.
- Session Management Function (SMF): Responsible for the interaction with the data plane and managing Protocol Data Unit (PDU) sessions.
- Unified Data Management (UDM): Stores the authentication data and subscription information and manages the authentication repository.
- Unified Data Repository (UDR): A database that stores and manages subscriber data, identities, and service configurations.
- User Plane Function (UPF): Function that manages all the functionality related to the user plane, enabling data forwarding and packet processing.

**Figure 5:**
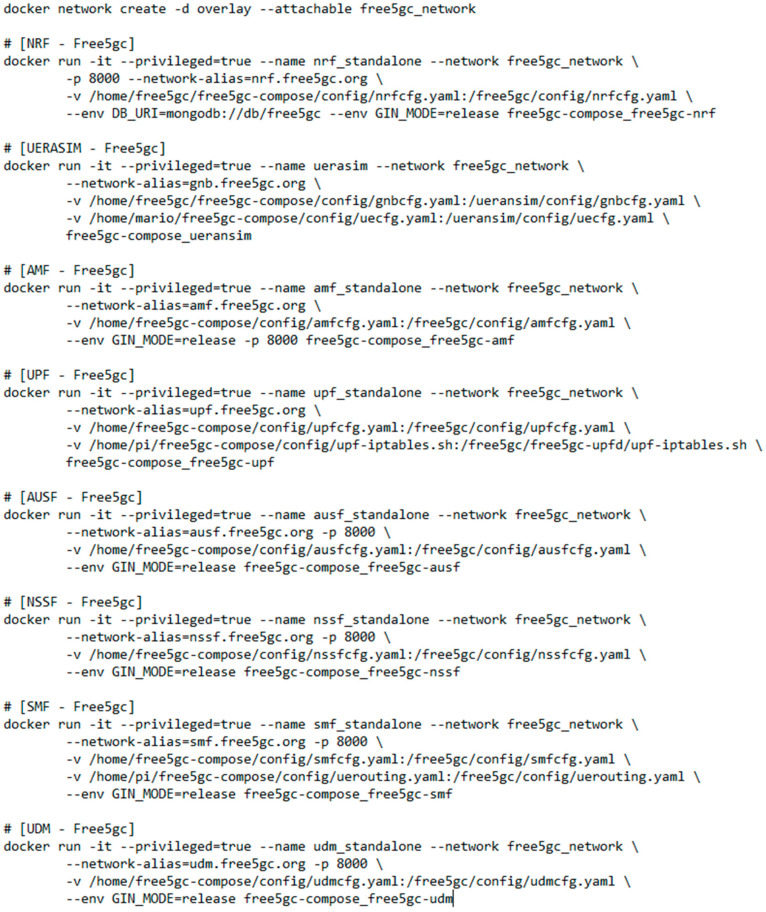
Example of distributed provisioning 5G Core via Docker.

The different NFs are implemented separately as stand-alone processes that interact using the standard defined interfaces. This means that the NFs can be deployed separately using virtualization or container technology. Figure 5 shows an example of the distributed deployment, using overlay networks, of the NFs associated with the free5GCore implemented in Docker.

#### 3.3.4. Network Monitoring Module

This module is responsible for monitoring the complete operation of all information exchanged within the NDT. Once all the DTs are provisioned and configured, the monitoring module will control the activation of port-mirroring functionalities within each one of the subnets present in the resulting NDT, with the aim of being able to use this information traffic for the generation of datasets that allow for the improvement of the complete emulation of the NDT.

#### 3.3.5. Traffic Generation and Injection Module

This module offers the option of enabling the traffic generation, based on different 5G traffic models over the NDT, and allows the validation and analysis of network performance.

The main goal of this module is to provide a way of injecting traffic in the 5G core network-virtualized infrastructure without dealing with actual Radio Access Networks and 5G-ready hardware. This module acts as a signaling NAS traffic generator that is able to communicate with the AMF NF in the 5G core by emulating the operations of User Equipment in a real environment. This module can perform session management, UE registering and de-registering, etc. Additionally, it can maintain GTP tunnels with a UPF in the core network and send data through them. The user data to be sent through this module, which acts as a broker, is captured in a network interface, and can have any virtual appliance or hardware machine as a source, as long as they are connected to the module interface.

Both the NAS signaling emulation (Signaling Traffic Generator—STG) and the users’ data broker (User Traffic Generator—UTG) are implemented in a single process, enabling the possibility of registering UEs, establishing PDU sessions, and sending user traffic to a data network from the same software tool, associating the information of the signaling traffic to the tunnel generated for the users’ traffic.

#### 3.3.6. Bidirectional Pipeline

Once the NDT infrastructure has been deployed and provisioned, it is necessary to establish the different pipelines to be used in the complete system. Depending on the type of elements interconnected and their communication necessities, two different pipelines are distinguished: V2V (virtual to virtual) and P2V (physical to virtual). The former is performed within the NDT, interconnecting each of the DTs that compose it. These pipelines allow for reflecting of the behavior of the communication that takes place in the physical world, without the time limitation present in the physical world, which allows the communication of large data flows in reduced times, and facilitates the emulation task, accelerating the obtention of results. The second supervises the interconnection between the virtual world of the NDT and the physical world, allowing the continuous feedback loop between the DT and its physical counterpart, providing the system with continuous co-evolution and cooperation capabilities.

Focusing on the type of P2V communication, it is necessary to approach it from a cybersecurity perspective due to the risks and threats associated with an environment of this type, such as risks associated with data, risk to physical devices modeled through DTs, to networks through those that are interconnected or to the network applications that operate on them. As described by Chen et al. in [29], P2V communication in NDT architectures must comply with the requirements of data confidentiality and integrity and the stability of the authenticity of policies, as well as the unforgeability based on the authenticity of both parties, physical and digital.

#### 3.3.7. AI/ML Module to Drive Smart Actions

This module will provide AI components, which are AI/ML models built (or imported) into the platform and deployed as smart agents. These smart agents can perform a variety of downstream tasks, such as optimizing network policies (e.g., traffic routing, resource deployment strategies, etc.) or predicting network failures. To that end, this platform will continuously monitor the NDT, providing the collected information to the smart agents in order to take appropriate actions to perform some arbitrary tasks. These changes can be replicated in the physical world through the P2V pipeline. It should be noted that the changes are not necessarily always replicated in the physical world, which is a more complex process that involves human verification to ensure that the changes are safe can be also considered. In Section 4, Section 5 and Section 6, we provide further details that expand the internals of this AI/ML module. More precisely, we will discuss the main AI components and applications that can be deployed in an NDT scenario.

### 3.4. Digital Maps: Preparing the Way towards an NDT-Ready Architecture

In order to tackle specific problems in the NDT scenario without requiring planning, designing, and implementing a complete NDT system from scratch, which would be very costly and time-consuming, the research community has begun to consider the possibility of building NDTs in a modular fashion. In this context, we propose to adopt the term Digital Map (emerged in recent IETF discussions) to describe a DMap as a subset of an NDT that focuses on a single specific dimension of the NDT functionality exposing its services through an API that other DMaps can easily consume to interact with it. The scope of application of a DMap is purposedly limited in nature; hence, it targets a narrow set of tasks, such as fault test simulation or energy optimization of the real network. The bidirectional flow of information between the virtual network and the physical system, formatted in JSON or another similar data exchange format, can synchronize the state of both networks. In this way, DMap can be incrementally developed, and the interconnection of several DMaps can lead to a complete NDT system.

**Figure 6:**
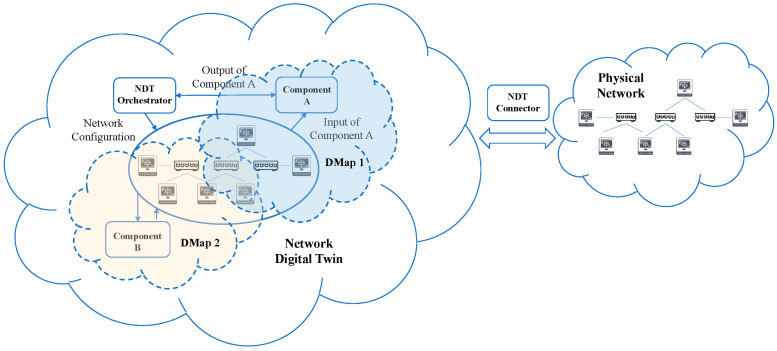
Diagram architecture of the DMap-based NDT.

In our proposal for a DMap-based NDT solution, which is illustrated in Figure 6, the following elements can be identified: (1) the physical network, (2) the Digital Twins (DTs) of the physical components of the network, (3) the NDT that represents the interconnection of DTs and comprises the virtualization of the network, the data flows between DTs and the DTs that conform it, and (4) several Digital Maps (DMap) that represent a set of functionalities build upon one or more DT, thus serving as a concrete view or dimension of the NDT. The rationale behind the Digital Map concept, which we propose as a novelty, is justified on the need to build a complete NDT in a modular manner and thus avoid the cost of planning, designing, and implementing a complete NDT system from scratch. In this way, DMaps considerably reduces the complexity of building a fully featured NDT system that completely replicates the physical system.

In particular, we propose a minimal NDT architecture that allows the connection of new DMap modules to an existing network to incrementally build an NDT system. The minimal architecture consists of an NDT connector, which is responsible for the communication between the NDT and the physical network, and an NDT orchestrator, which is responsible for managing and coordinating the orchestration of the different network elements that compose the NDT. In particular, the NDT connector serves as a bridge that enables data exchange between the virtual and physical networks. In this way, the data flow between the network components of the physical system and the corresponding replicas of these components (DTs) in the NDT are constantly synchronized. On the other hand, the NDT orchestrator will be responsible for replicating and provisioning the required network configuration of the physical system (network elements, interconnections, traffic routing, etc.). Based on this minimal NDT architecture, new DMaps can be developed and included in the NDT at any time to enrich it with new functionalities. It should be noted that multiple DMaps can share some of the underlaying components that conform the minimal NDT architecture (e.g., a subset of replicated network elements, the NDT connector, and the NDT orchestrator).

In addition, the deployment of a DMap-based NDT allows for selectively limiting the scope and visibility of the network according to the operator needs. Thus, a DMap can be deployed to cover a specific set of network components, or a specific range of IP addresses based on the specific service provider requirements. In contrast, a monolithic approach to the development of a DMap does not allow such flexibility and the NDT will always have full network visibility in all situations. That is, if a specific subnet must be excluded for some reason during network operation, it will be necessary to create a new NDT for that purpose or, alternatively, to mask it from the rest of the NDT, which may not be trivial and may require additional operational overhead.

We envision that a DMap-based architecture design can pave the way towards a complete NDT architecture, providing a cost-sensitive approach to incrementally build an NDT and delivering a flexible design to quickly adapt to the changing needs of the network. To that end, in order to make it easier for an NDT infrastructure to interact with the diverse ecosystem of 5G networks, standardization efforts are the first prerequisite to any successful implementation of such a system. We suggest that a standardization working group should focus on (i) providing a detailed description of the DMap architecture and the mechanism for DMap interconnection, (ii) define the DMap functionalities that need to be exposed through the DMap API, (iii) specify the structure of the messages exchanged between DMaps and the physical system, and (iv) determine the message exchange mechanism (protocols) between DMaps and the physical system. As a second prerequisite, we propose to consider the creation of a working group to develop a reference implementation of an NDT-ready architecture based on DMaps. To that end, the architecture proposed in Section 3 of this work can serve as the basis for the working group’s initial proposal.

Based on the normalization of these interconnections, we propose the establishment of the term “NDT-ready” to refer to a real system applying these standards. In this way, an NDT-ready label on a physical system implies that it could be connected to different NDTs that conform to the interconnection standard. Vice versa, an NDT that conforms the interconnection standard could be directly connected to any physical system with the NDT-ready label.

Moreover, an effortless integration of any DMap into the NDT-ready system can be achieved using the aforementioned interconnection standards. In this regard, we envision that task-specific DMaps can be offered as a service and seamlessly integrated into an NDT-ready system. In this way, a new disruptive business model can be created for the development, distribution and deployment of DMaps that extend the capabilities of commercial and industrial 5G networks to support the creation and deployment of emerging vertical services.

In Section 6.2, we will discuss the application of this innovative concept in a practical use case related to energy consumption optimization. In particular, we will emphasize the benefits of approaching this specific problem with our proposed architecture instead of the traditional way.

## 4. AI Applications in B5GEMINI

In the following subsections, we describe the innovative AI applications that B5GEMINI, as an NDT platform, will enable to deploy. In particular, we will discuss the potential of B5GEMINI as a platform to provide data for ML/DL model training. In addition, we will describe interesting applications related to network exploitation, where B5GEMINI has the potential to revolutionize the network management landscape, providing greater value and capability to network operators and service providers over traditional approaches. Next, we will explore the capabilities of B5GEMINI as a platform for distributed training of ML/DL models and inference tasks over the network. In the same vein, we will discuss the possibility of deploying pre-trained models over the NDT to perform arbitrary downstream tasks in an off-the-self fashion, which has the potential to improve convenience and enable cost reduction and flexibility for network managers and other stakeholders.

### 4.1. NDT for Smart and Robust-by-Design AI/ML-Based Applications

As it is well known, most ML algorithms need large amounts of real data to be trained. One of the major potential applications of an NDT is that it could serve as a platform for data collection that can be later used to train algorithms when the real network does not provide enough data, or it is too complex or expensive to gather. Moreover, there is an increasing need to design realistic virtual network scenarios in which different types of traffic can be generated in a controlled way and ML models can be trained and validated. B5GEMINI provides these capabilities, allowing the generation and capture of network traffic in a controlled manner to be used for training and testing ML components with realistic data. It should be noted that since the collected traffic is generated in a controlled environment, data labeling, which is required for training and testing supervised ML components, can be conducted without human intervention. This capability facilitates the labeling of data sets in the big data regime that are required to train and validate complex Deep Learning (DL) models. In addition, B5GEMINI replicates physical network elements and therefore, ML and DL components can be deployed and validated in real-time scenarios. Furthermore, using these approaches, different problems and faulty behaviors that rarely occur during the real network operation can be easily replicated now without negatively affecting the network. In this way, this approach allows one to train ML components for those situations in a more cost-effective and safe manner in order to ensure that they operate accordingly when they occur in real-world situations.

Moreover, recent applications of General Data Protection Regulation (GDPR) imply new challenges for the design of robust AI systems in the context of automated decision-making systems [30]. In this regard, interpretability is the key metric for providing reliable explanations of results, which is critical to avoid negative consequences of their use that may harm data subjects. To this end, these systems must provide not only a clear and intelligible explanation of their results, but also a way to assess the reliability of the results. At the network level, this interpretation capability must be technically accurate with respect to this domain to provide meaningful insight that can successfully explain the underlying decision-making process of the AI/ML system to network operators.

On the other hand, ML models are known to be vulnerable to adversarial attacks, which are attacks performed by adding small perturbations to the input data in a way that is unperceivable to humans but can trick a machine learning model into predicting an incorrect label, even with high confidence. To address this problem, ML resilience has emerged as a new research area that introduces mechanisms that make ML models more robust to adversarial attacks. In order to adopt a robustness-by-design approach, both Explainable AI and ML Resilience are two methodologies that should be carefully considered from the early stages of the ML/DL model design and development pipeline. Both are described in detail in Section 6.2.

### 4.2. ML for NDT Deployment, Configuration, and Monitoring

The dynamism of an NDT allows one to represent multiple realistic situations in a network: network connection topologies variability (e.g., ring or star architectures, redundant paths), location of specific functions (e.g., content caching, traffic filtering, hardware accelerators), network links capacity or technologies used, nodes and links status change (congestion, drop, or capacity variation), etc. Taking advantage of the NDT software configuration flexibility, different problems can be simulated and changes in the NDT instantiation introduced.

In this context, B5GEMINI ambition is to be able to use AI-based management for orchestration and dynamic control of the deployment of instances and components of the NDTs, which allow us to obtain the best results regarding the problem posed. In this regard, B5GEMINI can be used to verify SLA requirements in advance and adjust the strategy in time to reduce cost and uncover potential problems before the establishment of the service. For example, a 5G network slice can first be verified in an NDT by simulating the effects of user equipment, traffic, and network functionalities to uncover potential issues that may affect QoS. With this end-to-end verification process, the NDT can be used to ensure that the 5G service can be provided to the user with the expected quality and obtain the optimal strategy to optimize the resource to guarantee the QoS of the network slice throughout its lifecycle.

On the other hand, networking solutions have also evolved toward more software-oriented, cloud-based deployment. Software-defined networking (SDN), network functions’ virtualization (NFV), and cloud-based network management are being used by service providers to deploy new services faster, automate network management and operations, and improve network security. However, this has led to an increase in the number of software components and the dependencies between them, which in turn has increased the complexity of networks, making it difficult for network operators to get an accurate picture of the network. To provide network administrators with a clear understanding of the physical and virtual aspects of the network, B5GEMINI can be used to monitor in a dashboard the network in order to quickly identify and diagnose problems and take corrective action to improve network security and performance.

Finally, network operators can also use B5GEMINI as a platform to monitor and assess the impact of different 5G implementation strategies in highly dense and dynamic environments, while providing automated recommendations to improve network performance. Exploiting the data collected from the NDT, ML algorithms can be used to fine-tune these strategies to maximize utilization and optimize operational costs, thus serving as a valuable tool for network providers to accelerate 5G network rollout. Traditionally, this task has been performed by using simulation tools such as OMNET++, NS3, and NS2. However, these simulators require significant effort to create a realistic product environment and are only able to reproduce the 5G environment with a low degree of accuracy. In addition, these tools are only capable of one-way communications and therefore cannot take into account the dynamic nature of the real environment. This can lead to a significant desynchronization with the gathered data and the current situation of the real network. Moreover, simulation tools present a trade-off between complexity and fast response, reducing the potential for rapid iteration, which can severely affect the time to market. In contrast, B5GEMINI can provide a real-time assessment of the 5G network with little overhead, allowing for faster and more accurate assessment. In addition, with this approach, AI methods can be more easily integrated to gain feedback from the network configuration, provide suggestions to optimize it and enforce the configuration automatically in the live environment. In this way, B5GEMINI can serve as a key tool for network operators to manage the deployment of 5G networks in a closed loop fashion to ensure timely and efficient network operations.

Some other representative use cases in B5GEMINI are network path optimization for a service, network growth or reconfiguration based on traffic growth patterns, network problem root cause identification, or optimal mitigation of a network attack.

The metrics collected and the data generated in the three scenarios described within the NDT can then be used to train ML models. In addition, for some cases, such as Reinforcement Learning (RL), the ML model proposes changes to the NDT and once completed the result can be evaluated. In addition, the integration of ML can be used to forecast the future behavior of the network in order to identify early signs of problems ahead of time, which allow for better proactive network management. However, although what we described are the most prominent uses of NDT, it should be noted that these are not mandatory, i.e., network operators can use this technology to monitor NDT without considering the integration of ML for end-to-end optimization if it is not deemed necessary. In any case, the flexibility of the proposed architecture will make it possible to empower any NDT component with intelligent capabilities very easily at any time.

### 4.3. Distributed Training and Inference on Demand

We propose to use B5GEMINI as a platform to perform distributed training and inference processes for ML and DL models. The approach consists in virtualizing, instantiating, and allocating virtual GPUs on demand within the NDT to provision all the required resources to train and evaluate the models. The proposed approach can facilitate federated learning schemes and provide increased capabilities for the next-generation edge computing paradigm. It should be noted that to this day the virtualization process relies heavily on proprietary technology (see the case of NVIDIA and its industrial-grade GPUs). We consider this situation as the major challenge that may hinder the adoption of this technology at scale, and, for this reason, we look forward to the development of more open solutions to eventually materialize this concept.

### 4.4. NDT for ML as a Service

In the proposed architecture, it will also be possible to deploy off-the-shelf ML models in the allocated resources that are prepared to perform a specific task with a high degree of efficiency. We propose to realize this idea providing a catalog of pre-trained models for a wide variety of tasks, the users may select one of these models depending on their requirements to perform some downstream task on the NDT. For example, this approach will allow classification models to be deployed at specific locations in the network (routers, terminals, etc.) to filter traffic coming from certain subnets where congestion has been detected according to certain rules; thus, it will be possible to improve network performance by minimizing resource usage. In addition, once deployed, the models can be periodically retrained in a self-learning fashion to improve their overall performance and robustness over time. It is worth noting that this approach can be naturally extended to DMap as a Service by deploying off-the-shelf DMaps (e.g., Energy optimization for AI components or Synthetic Data Generators) instead of plain ML models to provide a more complete solution for network management.

## 5. B5GEMINI AI Components

The key role of the integration of AI components in B5GEMINI is to enable the deployment of smart applications at scale. In this section, we describe the most promising research opportunities that could help to achieve this goal. Additionally, we discuss the most important challenges that need to be addressed in order to realize the potential of AI in B5GEMINI.

### 5.1. Synthetic Data Generation

The generation of synthetic network and user data using Generative Adversarial Networks (GANs) will avoid the privacy violations that can be incurred in an NDT when using data collected from a real user and the lack of publicly available data for ML training and testing purposes. Moreover, in a current scenario where communications are increasingly encrypted, there is a tendency towards a situation where most real data cannot be used for ML training and NDT tasks because their content cannot be accessed. In this context, GANs will make it possible to generate synthetic data of sufficient quality to completely replace real data, improving security and avoiding privacy leakage.

Although recent works [31,32] demonstrate that it is possible to replicate the statistical distribution of real data features with high quality, future work should investigate new metrics that can (i) guide the convergence of the GAN during training toward high-fidelity data generation and (ii) measure data quality not only from a statistical perspective, but also considering to what extent synthetic data can completely replace real data in different tasks (e.g., to train ML without using real data). Using these distances, efficient stopping criteria for GAN training can also be investigated.

To demonstrate the benefits of this idea, in Section 6.2, we describe a use case that realizes this concept to implement and integrate a DMap in the NDT that provides an environment for generating synthetic telemetry data (e.g., VM logs, network traffic connections, etc.). This environment can be used as a data augmentation technique to increase the data available for training ML/DL models that are integrated into the network to improve their generalization and performance. This reduces reliance on real data, which can be expensive or difficult to obtain. In addition, it allows data to be exported securely to third parties without compromising the privacy of the data subject. Finally, this environment allows the creation of traffic models to better understand network behavior without the need to use real data, which may be susceptible to security issues and/or privacy regulations that prevent its use for this purpose.

### 5.2. Model Optimization

Deep Neural Networks (DNNs) training is often performed on very expensive hardware accelerators that lead to high energy consumption and carbon emissions [33]. This issue has been a growing concern in the AI community in recent years and has motivated the development of more efficient algorithms to reduce the environmental impact of ML/DL models. This set of techniques, which have been commonly referred to as Green AI, aims to fulfil this promise by reducing the energy required in the training and inference of ML/DL models. On this line, several Green AI techniques have been proposed, such as NAS, Quantization, Pruning, Low-rank Factorization (LF), and Knowledge Distillation (KD).

The development of novel Automated ML (AutoML) techniques in the context of NDTs will provide a mechanism to generate optimized AI and ML components that can be considered sustainable from an energy consumption point of view (Green AI principles). For example, to generate compact ML-based attack detectors to be deployed in resource-limited edge nodes.

Recently there has been an explosion of research interest in AutoML, with special attention on Neural Architecture Search (NAS) [34] that aims to generate a compact but robust and well-performing neural architecture by selecting and combining different basic components from a predefined search space. Furthermore, Google Brain introduced AutoML-Zero, an evolutionary meta-algorithm that generates a wide variety of compact machine learning algorithms for data classification [35]. This algorithm overcomes a well-known limitation of NAS, which greatly restricts its search algorithm (by selecting only neural networks). 

Nevertheless, the application of this technology is still in its infancy and several open problems [36,37] might limit its adoption in NDTs: (i) the majority of the techniques have been developed for computer vision and natural language tasks, (ii) it is not possible to interpret why an AutoML-generated model is better than its human-generated counterpart, (iii) robustness against adversarial data that can fool the model, and (iv) the lack of a complete AutoML pipeline to integrate these techniques in current ML development pipelines.

In addition to AutoML, another effective approach to realize Green AI is to use algorithms that allow for more power efficient ML/DL model development and deployment, either reducing the number of computations required for model training, compressing model representations using more efficient data structures, or finding ways to reuse computations. On this line, Pruning is a technique to reduce the size of a DNN by removing unnecessary weights [38]. Pruning can be performed manually (using a manually designed heuristic approach to remove unnecessary weights) [39] or automatically (using algorithms to automatically remove unnecessary weights from a DNN) [38] or a combination of both [40].

Moreover, Quantization is a technique that allows for DNN models to be trained with lower precision, which can lead to improved efficiency [41]. Stochastic Quantization has been successfully applied to convert gradients to lower bit width representations during the backward pass; this enables the use of bit convolution kernels during the backward and forward passes, which further accelerates training times and speeds up inference times [42]. In addition, Quantization has also been applied in the form of a differentiable non-linear activation function to reduce training times [43]. In this way, the Quantization is learned in a lossless and end-to-end fashion during the training. This method is suitable for arbitrary bit width Quantization and can be applied to both weights and activations of the DNN.

On the other hand, LF is a technique for representing a matrix with a smaller number of parameters. This can be conducted by decomposing the matrix into a product of two lower-rank matrices. Taking advantage of this concept, LF has been applied to reduce weight matrices that represent ML/DL model parameters [44]. LF has also been applied to training dataset compression [45,46]. In NN, the number of neurons in the input layer depends on the size of the feature space. Reducing the dimensionality of the feature space, the size of the DNN is also reduced. To achieve this reduction in dimensionality, input data in the form of matrices are factorized into low-rank matrices to adjust to the hardware characteristics in an automated fashion. In this way, both the in-memory size of the DNN and the inference time are reduced, while the accuracy is preserved.

On the other hand, KD is a technique for transferring the knowledge learned by a large DNN into a smaller one. This can be conducted by training the smaller DNN to mimic the predictions of the larger one [47]. KD, which was first introduced by [48], has been successfully applied to compress large transformer models such as BERT (distilBERT) [49].

Prior to ML/DL model optimization, it is imperative to first establish accurate methods to estimate the energy cost associated with training or inference tasks. Several studies have focused on optimizing ML-specific models by adopting general estimation models that characterize energy consumption (either at the software or hardware level) to the ML domain.

In the use case shown in Section 6.2, we apply these techniques to automatically search for an optimal model configuration from an energy consumption perspective. Specifically, we propose to introduce the energy consumption factor of the model during the NAS procedure. For this purpose, we propose to design a model that allows estimating the energy consumption of each model during the inference operations. Thus, by introducing this variable in the NAS procedure, it is possible to automatically find DNN architectures that satisfy arbitrary metrics that jointly consider model performance and energy efficiency. Furthermore, in the presented use case, the application of Quantization, Prunning, LF, or KD techniques is also possible in order to compress the model representation with the ultimate goal of reducing its memory footprint and power consumption while minimizing accuracy loss.

### 5.3. Model Robustness: Resilient and Explainable AI

To achieve a robustness-by-design approach to build safe and reliable ML/DL models for production-ready applications that we described in Section 4.1, in this section, we present the concept of explainable AI, which encompasses a series of methods to achieve interpretable models or post-hoc explanations that ensure transparency and accountability of the system in its decision-making process. Furthermore, we describe some of the ML resilience libraries and toolboxes that have been proposed in the literature to secure ML models against adversarial attacks and how they can be applied to B5GEMINI to achieve robust ML models.

#### 5.3.1. Resilient ML

Recent findings have demonstrated that ML models, DNNs in particular, are vulnerable to malicious inputs, called adversarial examples, that are modified to spoof the ML algorithms and cause them to yield erroneous outputs [50]. Therefore, it is crucial that resilience to these sophisticated attacks is added to ML algorithms to minimize the current resistance to the adoption of ML components in industrial environments. For this purpose, B5GEMINI will integrate existing adversarial libraries and toolboxes (e.g., CleverHans [51], DeepRobust [52], Adversarial Robustness Toolbox [53]) to provide resilience to ML components within the NDT.

Nevertheless, the existing defense mechanisms are limited because robustness against specific attacks needs to be provided in specific settings. The design of a robust machine learning model against all types of adversarial examples is still an open research problem.

#### 5.3.2. Explainable ML

Another key aspect of network operation is the necessity to understand the intentions and guarantees of the applied policies and the possibility to extract knowledge about failure cases when they occur. For this reason, network policy explainability also needs to be considered in order to help with stakeholder buy-in for real-world applications. Moreover, the ML model interpretability is of extreme significance to explain the results obtained from the envisioned models and help to distinguish adversarial from legitimate inputs.

Several state-of-the-art solutions have been proposed to achieve explainability in ML:

1. Local Interpretable Model-agnostic Explanations (LIME): is a technique proposed by Ribeiro et al. to provide local explanations to the predictions made by black-box models [54]. This is performed by approximating the model prediction with a simpler surrogate model that behaves in a way that is easier to interpret. This technique has proven effective for a variety of ML models, including DNN.
2. Shapley Values: is a technique that can be used to explain the contribution of each individual input to the final prediction of a ML model [55]. They were first proposed by Lloyd Shapley in 1953 as a way of calculating the contribution of each player to the success of a game [56]. In this way, Shapley Values can be used to calculate the contribution of each input to the prediction of a ML model, as well as the contribution of each layer in a DNN.
3. Partial Dependency Plots (PDG): is a technique, first proposed by Friedman et al. in 1990 [57], that can be used to visualize the relationship between the inputs and output of a ML model. PDP can be used to visualize the impact of each input on the prediction of a ML model, as well as the impact of the interactions between inputs. PDP has been later extended by the technique of Individual Conditional Expectation (ICE), proposed by Goldstein et al. [58].

However, what all these methods have in common is that they are computationally expensive and are not applicable to all models. Explainability remains as a difficult challenge, severely compounded by the need for these algorithms to also be accurate and unbiased. Overall, this is an exciting area of research that will undoubtedly receive increased attention in the coming years.

### 5.4. Novel Deep Learning Architectures

Graph Neural Networks (GNN) are neural network architectures able to process graphs as inputs to produce useful embeddings exploiting the concept of message passing between nodes in the graph [59]. GNNs have yielded groundbreaking results in many fields where data is fundamentally structured as graphs, such as the field of communication networks, where this innovative type of neural networks is deemed as a key technology to model complex graphs (e.g., traffic routing optimization, resource allocation, anomaly detection). Due to their unique capabilities over traditional neural networks, we envision GNN as a key player in the construction of NDT for communication network modeling. Several studies have demonstrated that GNNs are able to remain robust to changes in network topology, routing policies and traffic distribution [60], a key advantage over alternatives such as traditional neural networks (e.g., recurrent neural networks, convolutional neural networks, variational autoencoders) or Deep Reinforcement Learning techniques that are severely deprived of this capacity [61]. However, GNNs present a limiting factor due to weak scalability, which inevitably must come as a trade-off that sacrifices graph completeness, either losing node neighbors when using a graph sample strategy or structural information when opting for a graph clustering method [59], ultimately resulting in lower performance in either form. On the other hand, in convolutional graph networks (GCN), one of the most successful GNN architectures, the graph convolution operation can be interpreted as a Laplacian smoothing. It has been demonstrated that repeated application of Laplacian smoothing on the same graph leads to the convergence of all node embeddings to the same value [62]. This result leads to the conclusion that stacking multiple convolutional layers will not always result in a more powerful model; in fact, it has been demonstrated that the performance of GCNs significantly decreases as more layers are added [62]. This finding makes scalability of GNNs an even more concerning problem. For the above reasons, we consider this issue as the most important challenge to be solved for the adoption of building NDTs.

In the field of communication networks, DRL has recently received more attention because it is emerging as a natural candidate to effectively address various problems and challenges intrinsic of this domain. Network devices need to make autonomous decisions to achieve different objectives or meet QoS requirements. Most of the decision-making problems in network optimization scenarios can be formulated using Markov Decision Processes (MDP). Reinforcement Learning (RL) techniques have been widely adopted to solve MDP problems [63]. However, RL techniques are not always applicable, especially when the problem is too complex, or the state and action spaces are too large. Deep Reinforcement Learning (DRL) has been demonstrated to be promising in these cases. DRL is a subfield of RL that uses DL techniques to allow machines to learn how to achieve a certain goal by trial and error, without the need of labeled data. More precisely, a DRL agent is trained by interacting with an environment, and the environment returns a state and a reward after each action. The first phase of the training process is composed of two main parts: first, the DRL agent collects all the states and rewards from the environment, and second, the DRL agent’s past experiences are used to train the weights of a Deep Neural Network (DNN) in a supervised manner. Similar to GNNs, DRL-based algorithms often suffer from poor scalability. The reason is that, as stated above, the DRL agent learns from sequential interactions with the environment. For that reason, when DRL is applied to large networks or complex optimization problems when the interaction with the environment occurs slowly, the problem becomes computationally expensive. Solutions have been proposed to accelerate the DNN weight update stage using Evolution Strategies (ES) [64]. Nonetheless, solutions to accelerate the interaction with the environment remain unclear. Some proposals, such as Distributed RL or asynchronous methods, try to reduce the number of interactions between the agent and the environment in order to speed up the learning process, but the problem with these approaches is that the agent might not learn as efficiently as with a more traditional synchronous approach.

From a more pragmatic perspective, we describe below some interesting use cases of GNN in the networking landscape, which can be leveraged to facilitate the deployment and management of 5G and beyond networks. To being with, some proposals have focused on modeling complex topological relationships that allow for generating useful representations to predict latency parameters such as delay, jitter, and packet loss for the different potential routes that can be considered in the transmission of data from one node of the network to another [60,65]. The objective is to use this information to apply an optimization process that allows for the automatic planning of the routing of traffic in a network considering the different qualities of the service. Furthermore, this technology can also be applied to find the optimal links that should be placed between the different nodes of the network to reduce those latency and loss metrics.

In addition, one study has used a combination of RDL and GNN to predict the optimal policy for the placement of VNFs in a dynamic network topology (i.e., nodes and links that can appear and disappear at any time), based on the resources required by the VNFs (e.g., memory and CPU) and certain QoS constraints (e.g., latency, bandwidth, packet loss, etc.) imposed for each packet flow [66]. In this context, due to the intrinsic dependence on the specific network structure of RDL-based methods, the incorporation of GNN helps to achieve generalization across network topologies, which is crucial to address the dynamic and heterogenous nature of 5G and beyond networks.

On the other hand, another study proposed to use a Spatio-Temporal Graph Convolutional Network (ST-GCN) over the data plane of an SDN to map the network into a graph structure [67]. In this way, the ST-GCN can learn from certain features of the packet flows to generate a network representation that is fed to a DNN to obtain a classification by flow (normal or attack). The method can also provide information about the path that DDoS attacks follow through the network, facilitating DDoS mitigation.

In another study, a GCN with Gated Recurrent Unit (GRU) cells was used to predict the state of links in a network [68]. In particular, the GCN was used to learn features of the network topology, while the GRU was used to model the temporal dependencies between link states. The model was trained with a dataset of link state characteristics (bandwidth, delay, and packet loss rate) and was able to predict the state of links in the network with lower error rates than other state-of-the-art methods such as LSTM-based models.

Finally, the deployment and chaining of VNFs is a highly complex problem that can be formulated as a graph optimization problem. Some studies have proposed using a GNN to model the problem as a graph and find the optimal deployment of VNFs and the optimal sequence of VNFs for a given service request chain (SRC), while also considering the energy consumption of VNFs in the problem formulation [69,70,71,72,73]. In fact, the complex management and orchestration processes involved in 5G/6G networks are susceptible to being represented as graphs, opening up the possibility of applying GNNs to provide a high degree of intelligence to automate these processes.

### 5.5. Uncertainty Estimation

In a general sense, Neural Networks (NN) decisions are inherently unreliable because they lack expressiveness and transparency. A NN is a stack of continuous geometric transformations that are learned to map one vector space into another to minimize some objective functions. In simple terms, a NN cannot understand or resonate about the content of the data being trained on, which leads to their inability to explain its decisions, as well as their sensitivity to small changes in the data distribution, making it difficult to rely on their predictions. Additionally, NN are often overconfident in their predictions and vulnerable to adversarial attacks [74]. To address network problems, reliable solutions are a fundamental need for network operators, as they can have a significant impact on service quality and customer satisfaction. There have been some proposals based on ensembling and Bayesian learning to endow this capacity to NN. Using these techniques, probabilistic confidence values can be associated with the NN output. In this way, the reliability of NN predictions can be estimated to provide end users with more realistic decision making. A comprehensive review of these methods is provided by Gawlikowski et al. [74].

### 5.6. Intent-Based Networking

Intent-based networking is an approach to network operation that focuses on user intent rather than network details. In an intent-based network (IBN), operators can define the desired objectives or outcomes in a declarative manner and then rely on the network to automatically provision all the required resources and configure itself to achieve those objectives, ideally, in an optimal manner. This contrasts with the more traditional approach of configuring a network by manually specifying all the required configuration parameters. 

Intent-based networking is particularly well suited to manage large, complex networks because it greatly simplifies operation tasks by automating highly specialized and time-consuming processes such as network configuration and troubleshooting, ultimately reducing the time and resources required to manage the network and making it easier and quicker to deploy new applications. In addition, by reducing manual interactions with the network, IBNs can also help reduce the risk of human error. IBN is also a valuable tool for NDT management, as the same advantages can be transferred to this environment, where the need for manual setup and maintenance is greatly reduced, and operational efficiency is increased.

Software-Defined Networking (SDN) and Network Function Virtualization (NFV) are two key enablers of IBN. NFV and SDN are complementary technologies that are often used together to provide a more complete IBN solution. NFV decouples network functions from dedicated hardware devices, allowing them to be run on standard server hardware. This greatly increases the flexibility of the network, as different functions can be quickly and easily deployed, reconfigured, or upgraded as needed. SDN, on the other hand, provides a centralized control plane that can be used to manage and configure the network. Together, NFV and SDN allow for the creation of virtualized networks that are much more flexible and easier to manage than traditional networks [75].

IBNs are based on the principle that guides Infrastructure as Code (IaC). In this context, the network is treated as a software system, and is configured and managed by code. Driven by this conceptual analogy, one approach to achieve an intent-based network is to create a Domain Specific Language (DSL) to formally specify network policies. This DSL can be used to define the appropriate configuration of network devices and traffic routes. This configuration, often referred to as a “policy” in this context, is defined as a sequence of actions to be performed on the network, which can be formulated as a Markov Decision Process (MDP).

There are still some challenges to be solved before IBN can be fully realized. Chief among them is the need for more intelligent algorithms that can automatically and reliably translate high-level user intentions into a low-level network configuration policy that copes with the ambiguities and lack of proper context that human language often presents. In addition, these algorithms must take into account the subtle conflicts and incompatibilities in the execution of each user intent and be able to automatically resolve them or suggest a possible resolution to the network operator [76]. Currently, there is no clear consensus on how to solve these problems in a scalable and efficient manner. For this reason, although IBNs have been widely recognized as a promising paradigm for future network operation, the lack of efficient methods for policy inference limits their applicability. Further research is needed to enable the widespread adoption of this concept.

## 6. B5GEMINI Use Cases

In this section, we describe two use cases to demonstrate the advantages of using the proposed NDT architecture. In the first use case, we propose to develop security capabilities that help detect malicious elements in the real network in order to prevent or mitigate potential cyber-attacks in a 5G cloud infrastructure. In the second use case, we propose the realization of energy optimization mechanisms for NDT integrated AI components that can be seamlessly deployed in the real network in a secure and reliable manner.

### 6.1. Cyber-Attack Use Cases on Cloud 5G Infrastructure

One of the most relevant issues in 5G and beyond is security. The evolution towards distributed solutions in the cloud, with multiple providers and hyperscalers, can represent a handicap at the security level for future 5G services, since the network operators will not have absolute control of the infrastructure. Analyzing the capacities and performance of AI-based solutions to address attacks to the cloud environments represents an interesting topic. Using an NDT with 5G functionalities, based on NFV and cloud architectures, allows one to introduce attacks in the virtual environment without causing harmful situations on the physical system and related 5G services. In this way, this approach serves as a foundation to enable advanced AI-based solutions.

On this basis, some cybersecurity attacks are proposed with an NDT 5G cloud environment with generalized encryption (at signaling and data plane levels). A first case to be evaluated in the NDT is an attack on the illegal consumption of resources due to the presence of malware. The cryptomining variant of malware (intensive use of CPU and memory resources for cryptocurrency mining) is a lucrative criminal activity today, impacting the performance and cost of certain cloud services. Analyzing and classifying the traffic that is generated, with AI models in an NDT environment, allows the creation and evaluation of tools to block this activity. Another case is the attack to the DNS, a critical service in 5G cloud infrastructure. Today, there is a progressive adoption of DoH protocol (RFC 8484), with a secure HTTPS layer for DNS resolution to increase user privacy. Precisely, the activation of this protocol variant in an NDT opens the possibility to study how flooding or other attacks variants affects the cloud infrastructure and provides a mechanism to detect the attack using AI.

In terms of Cloud 5G solutions deployment, we can consider several scenarios: native cloud, on-premises, or hybrid solutions (i.e., on-premises but integrated into cloud management to address regulatory aspects). In relation to the two-way communication capabilities that an NDT provides, and the information exchanged between the two parties (the NDT and the physical system), it should be noted that management systems support templates to automate deployments and simplify replications. These templates can be used as the automatic system input feed to the NDT (Section 3.3.1) for initial deployments of the NDT, on a reduced scale. For example, the 5G component, such AMF or UPF, can be deployed with few resources to manage reduced user traffic volume. Additionally, hyperscalers and on-premise setups have the capacity to deliver rich information based on supported API, including telemetry information (activity loads, traffic volume, etc.). This information can be understood as part of P2V pipeline input (Section 3.3.4), which will enrich the NDT behavior, such as statistical traffic profiles and behavioral patterns for the traffic generation (Section 3.3.5) and combine with cryptomining and DoH-related attacks. The NDT results will provide a customized AL/ML output (Section 3.3.6) to deploy again using the P2V channel. 

Nothing limits a dynamic interaction between real Cloud 5G Core and its NDT over time to improve the ML detection. This is possible because most of the workload falls on the side of the NDT (rebalance traffics generation types, attacks patterns, and re-train ML models), and avoids affections to production networks loads and costs that are focused on providing the 5G service. One potential advantage in the cybersecurity case for NDT is that we can limit the P2V data feed to only one direction of communication (from the real network to the NDT). The other direction feed (ML model updates), which implies changes in the production cloud, can be performed through the secured pre-existing management channels (i.e., through the provisioning system). This is also relevant when NDT evaluates different technicalities in the types of attacks selected (e.g., use DoH for data extra filtration, for malware control channel communication, or to launch packet flooding) because there is no risk to impacting the production network or rendering it inaccessible.

### 6.2. Digital Maps for Energy Optimization of Network AI Components’ Use Case

To demonstrate the advantages provided by the architecture proposed in Section 4.3, we present a use case for energy optimization that builds on the DMap concept to optimize the energy consumption of an NDT system by managing its resources. The need for energy consumption has been increasing in communication networks, as they have become more sophisticated and widespread over the years. To cope with the high energy demand, network providers must be able to manage their energy resources more efficiently. ML has proven to be a valuable tool to achieve this goal [77]. The same can be applied to an NDT scenario by using ML methods to optimize the energy usage of the grid by predicting when and where energy usage is highest. In this way, the network can be reconfigured to reduce energy consumption in those specific areas. In this regard, considering that AI components play an increasingly important role in current networks assisting other components in complex decision processes, it can be interesting to use an NDT to optimize their training and inference processes to optimize the energy consumption of the network. In this context, these optimizations can be applied first in the NDT and then seamlessly reproduced on the physical system after validation. With this innovative approach, optimizations can be performed in a more controlled and safer way.

**Figure 7:**
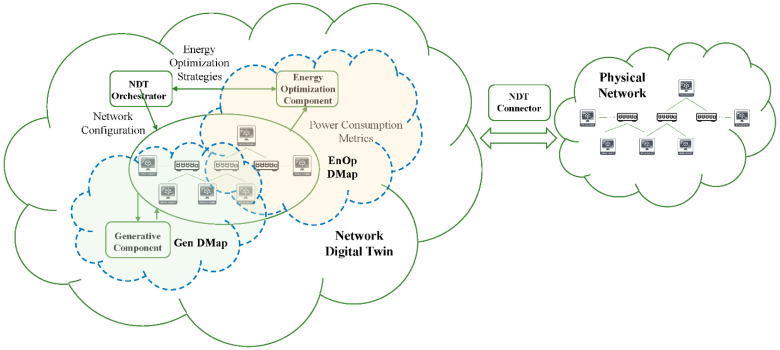
An illustration of the DMap-based NDT solution for the energy optimization use case.

In particular, this use case will illustrate how a physical network can be easily attached to a DMap that will support the optimization of the power consumption of the AI components of the network in a controlled manner. In addition, this use case will consider the implementation of a complementary DMap for the generation of synthetic data that can be used during training processes to boost the performance of ML components in the NDT or for exporting such synthetic data to be used by third parties without creating any privacy breach. We schematically represent the described use case in Figure 7.

We will develop EnOp, a DMap responsible for the energy optimization of the AI components integrated in the real network. The EnOp DMap will be obtained incorporating an Energy Optimization Component (EOC) in the minimal NDT architecture. The EOC component will allow one to optimize ML/DL models in order to reduce their power consumption in their training procedure and in downstream inference tasks over the network. The EOC component will be composed of an energy estimation model and an upgradeable collection of algorithms for optimizing ML/DL models. The energy estimation model will be in charge of defining the energy consumption of the ML/DL models, which can be conducted either by measurements on real hardware or using simulated environments [78]. Based on this power state, several algorithms can be applied to optimize the ML/DL components to adjust the power consumption of the network with minimal impact on its performance. In particular, we consider introducing the energy dimension in the automatic search of efficient DNN architectures using NAS. In addition, other techniques such as Quantization, Pruning, Low-rank Factorization, or Knowledge Distillations techniques can be also applied to compact the ML/DL models in order to decrease their memory footprint and accelerate training and inference task.

In addition, this use case proposes Gen, a complementary DMap for the generation of synthetic telemetry data (e.g., VM logs, network traffic connections, etc.). The Gen DMap will allow one to generate synthetic data that statistically replicate data of the real system with high fidelity. The synthetic data in combination with the real data can be highly beneficial for the training of ML/DL models because it can serve as a data augmentation technique to improve the performance of these models. Furthermore, the Gen DMap can provide a safe way to export data to third parties involved in cross developments without violating the privacy of data or incurring data breaches. Finally, the synthetic data could be also used to build realistic traffic models for a better understanding of the physical twin behavior in different scenarios without compromising user privacy.

The creation of a DMap that serves as a data source for the ML/DL model training and behavior modeling tasks is a promising way to circumvent the current obstacles to data processing and exploitation in real networks. It provides a network-aware and privacy-preserving data collection and management system that is critical to the proper operation of the AI/ML models. More importantly, the proposed solution is also easily scalable and portable, as it can be deployed in any NDT-ready network in a plug-and-play manner.

## 7. Conclusions

This article has presented B5GEMINI, an NDT for B5G networks that has the potential to serve as a platform for developing and deploying innovative intelligence-driven network applications, and we have also discussed the key role of AI in the construction, deployment, and operation of it. 

Current NDT proposals apply AI to optimize network management and orchestration processes in a controlled scenario that replicates the real network without compromising its performance and security. B5GEMINI goes one step further, focusing on the design, training, and testing of the AI components deployed in a real network. In addition to these two scenarios, distributed training and inference on demand, ML as a service and educational applications such as cyber-ranges can benefit from B5GEMNI capabilities.

To effectively enable all these scenarios in a realistic NDT, we have proposed B5GEMINI as an approach for a modular and scalable NDT system that uses DMaps, a novel design abstraction based on the recently appeared Digital Map concept. The introduction of the Digital Maps in the NDT architecture is fundamental to allow an iterative and incremental development of the same, avoiding a building-from-scratch approach and allowing the integration of new functionalities in a continuous and evolutionary way, which reduces the risk of errors in the planning process and optimizes the investment of resources throughout the development life cycle.

We have described the architecture of our system and presented all the interconnected modules that comprise it, spanning from the infrastructure level to the AI applications. The 5G core functionalities were integrated in B5GEMINI, adapting some of the NFs available in the free5GC project. Importantly, the proposed architecture has been designed to enable a plug-and-play experience that allows fast and seamless connection of NDTs with existing and new networks. We call this feature NDT-ready and it consists of verifying that a real network is compatible with standard NDT interfaces, thus ensuring interoperability with NDTs. In this regard, we have proposed, as future work, to promote the standardization of the interfaces of the hardware and software components that comprise the described architecture to guarantee interoperability between the NDT and the physical networks and thus allow for easier and wider adoption of this technology by the networking industry. 

Furthermore, we have detailed the main components that will be part of the AI ecosystem in B5GEMINI. Synthetic data generation, model optimization, and robustness, to name a few, are some of the key components that will enable the implementation of privacy-aware, efficient, reliable, and scalable AI-based solutions for end-to-end management of B5G networks in a safe and controlled manner. We envision that the described AI components present great potential to shape the NDT landscape in the near future. 

Finally, we have described two prominent use cases that highlight the capabilities of B5GEMINI and demonstrate the advantages it offers over traditional network approaches. In the use cases presented, AI components play a key role in enabling the efficient use of network resources and improving network performance, while reducing operational costs. In particular, we have applied some of the described AI components to solve specific problems within the NDT in a safe and controlled manner without compromising the security and performance of the real network. The first use case is related to the cybersecurity domain and focuses on the training and testing of ML-based cryptomining attack detectors. The second use case proposes the application of B5GEMINI to solve the energy optimization problem of ML/DL models deployed in the real network by leveraging the DMap concept to implement this functionality in a modular fashion over the proposed architecture, which lays the foundation to incrementally deploy additional layers to design and integrate custom AI-based applications to solve further specific use cases in the future.
